# Interspecific delimitation and relationships among four *Ostrya* species based on plastomes

**DOI:** 10.1186/s12863-019-0733-0

**Published:** 2019-03-12

**Authors:** Yanyou Jiang, Yongzhi Yang, Zhiqiang Lu, Dongshi Wan, Guangpeng Ren

**Affiliations:** State Key Laboratory of Grassland Agro-Ecosystem, School of Life Sciences, Lanzhou University, Lanzhou, Gansu People’s Republic of China

**Keywords:** Chloroplast genome, Interspecific relationship, *Ostrya*, Phylogeny, Species delimitation

## Abstract

**Background:**

The genus *Ostrya* (Betulaceae) contains eight species and four of them are distributed in China. However, studies based on limited informative sites of several chloroplast markers failed to resolve interspecific delimitation and relationships among the four Chinese species. In this study, we aimed to use the whole chloroplast genomes to address these two issues.

**Results:**

We assembled and annotated 33 complete chloroplast genomes (plastomes) of the four Chinese species, representing 17 populations across most of their geographical distributions. Each species contained samples of several individuals that cover most of geographic distributions of the species. All plastomes are highly conserved in genome structure and gene order, with a total length of 158–159 kb and 122 genes. Phylogenetic analyses of whole plastomes, non-coding regions and protein-coding genes produced almost the same topological relationships. In contrast to the well-delimitated species boundary inferred from the nuclear ITS sequence variations, three of the four species are non-monophyletic in the plastome trees, which is consistent with previous studies based on a few chloroplast markers.

**Conclusions:**

The high incongruence between the ITS and plastome trees may suggest the widespread occurrences of hybrid introgression and incomplete lineage sorting during the divergence of these species. In addition, the plastomes with more informative sites compared with a few chloroplast markers still failed to resolve the phylogenetic relationships of the four species, and further studies involving population genomic data may be needed to better understand their evolutionary histories.

**Electronic supplementary material:**

The online version of this article (10.1186/s12863-019-0733-0) contains supplementary material, which is available to authorized users.

## Background

*Ostrya* is a small genus belongs to the birch family Betulaceae and merely consists of eight species native in southern Europe, southwest and eastern Asia, and North and Central America [1–3]. These small deciduous trees, commonly called as hophornbeam or ironwood, are well known for their hard and heavy woods. *Ostrya* woods have been used for various purposes such as furniture, axles, fuel wood and charcoal [2, 4]. It had been long thought that there are five species distributed in China (http://www.efloras.org) until Lu et al. (2016) [5] found that *Ostrya yunnanensis* was nested within *O. multinervis* in both phylogenetic trees and morphological clustering. The two species were therefore recognized as the same species and renamed as *O. chinensis* [6]. Among the four species, *O. japonica* is mainly distributed in China, Japan and Korea, while the remaining three species, *O. rehderiana*, *O. chinensis* and *O. trichocarpa*, are endemic to China. The amount of these wild plants has been decreased rapidly due to overexploitation, habitat destruction and ecosystem deterioration. The famous endangered species, *O. rehderiana*, has been reported that only five mature trees survive and are conserved in the Tianmu Mountain, Zhejiang [7]. Although *Ostrya* species own excellent wood and are regarded as ecologically and economically valuable foundation for future forests, the interspecific delimitation and relationships remain unresolved. Previous study failed to discern the four *Ostrya* species based on four chloroplast (cp) fragments (*rps16*, *trnG*^(UCC)^ intron, *trnH–psbA*, and *trnL–trnF*) while the ITS sequence variations did [5]. It has been suggested that the traditionally accepted cpDNA barcodes own limited resolution in delimitating closely related species due to the insufficient informative variations [8–10]. By contrast, the complete chloroplast genomes (plastomes) were proven to provide more valuable information in revealing phylogeny of plants [11–16]. And with the development of Next-generation sequencing technology, it has recently become cost-effective and it is easier to sequence the complete plastomes in plants than before [12, 17, 18]. The plastomes with more informative sites may increase resolution of the previously unresolved phylogenetic relationships based on a few cpDNA markers [15, 19]. Therefore, in this study, we re-investigate phylogenetic relationships of the four Chinese *Ostrya* species based on plastomes. We selected multiple individuals per species to cover the most of geographic distributions of the species to reduce the sampling bias. We also extracted three datasets, i.e., the whole plastomes, non-coding regions and protein-coding genes, for phylogenetic analyses and compared these plastome trees with the ITS tree. Specifically, the aims of our study are to: 1) investigate whether the three plastome datasets result in consistent phylogenies and whether they can discern the four species and 2) test if phylogeny based on plastome datasets congruent with the ITS tree.

## Methods

### Plant materials, DNA extraction and sequencing

We chose in total 33 individuals from 17 populations representing all the four *Ostrya* species that occur in China. For each species, we selected samples that cover most of its geographic distribution (Additional file 1: Table S1). The fresh leaf materials were collected in the field and preserved with silica gel immediately. Total genomic DNA was extracted from 20 mg silica gel-dried leaves on the basis of the modified CTAB procedure [20]. According to the basic protocol, we prepared the end-repaired, phosphorylated and A-tailed DNA fragments and ligated with index adapters. The library construction and whole-genome sequencing were accomplished at Beijing Genomics Institute (Shenzhen, Guangdong, China). The libraries were sequenced on the Illumina Hiseq 2000 platform.

### Genome assembly and annotation

Raw reads of all samples were trimmed with a filter standard (Q < = 5 or N base content > 10%). Afterwards, we used Trimmomatic [21] to further filter the data. We downloaded about 3000 published chloroplast genome sequences from NCBI and built an index of them. All the high quality reads were then mapped to the index using Bowtie2 [22] and sorted by Samtools v.1.2 [23]. We used the software bam2fastq (https://gsl.hudsonalpha.org/information/software/bam2fastq) to generate the fastq format files from the bam file. Then, we assembled the plastomes using Velvet v1.2.10 [24] and filled up the gaps by GapCloser v1.12 (https://sourceforge.net/projects/soapdenovo2/files/GapCloser/). Moreover, we used Plann v.1.1.2 [25] to annotate the plastomes using *O. rehderiana* as a reference. Then we corrected the start codons, stop codons and intron/exon boundaries manually using Sequin v.15.10 (http://www.ncbi.nlm.nih.gov/Sequin/) as well as a visual software Geneious v.R.8.1.4 [26]. Ultimately, the circular images were performed through OGDRAWv.1.1 (http://ogdraw.mpimp-golm.mpg.de/) [27].

### Genome comparison and genome repeat

In order to show interspecific variations, the alignments of all plastomes were plotted using the mVISTA program with LAGAN mode [28]. All validated plastome sequences were submitted to GenBank (Additional file 2: Table S2). We determined three kinds of repeats among plastomes of the four *Ostrya* species: dispersed, palindromic and tandem repeat. The former two types were visualized and located by an online program REPuter [29] with a minimal length of 30 bp and 90% sequence identities (Hamming distance equal to 3) between the two repeats [30]. Tandem Repeats Finder [31] was used to detect tandem repeats with the following parameters: Match 2, Mismatch 7, Delta 7, PM 80, PI 10, Minscore 50, MaxPeriod 500, with similarity 100%. We further verified all the repeats and removed the redundant segment manually.

### Phylogenetic analysis

According to Grimm & Renner (2013) [32], the most closely related genus *Carpinus* could not be separated well from *Ostrya* and there were no published *Ostryopsis* plastome sequences available in GenBank. We therefore downloaded a plastome of *Corylus chinensis* (GenBank accession number: NC_032351.1) from GenBank as outgroup to reconstruct the phylogenetic tree of the four *Ostrya* species. In order to test whether different plastome regions resulted in consistent phylogenies during the phylogenetic reconstructions, we used three plastome datasets: (1) the whole plastomes, (2) the non-coding regions and (3) the protein-coding genes (PCGs). In addition, we downloaded from GenBank the same ITS dataset as in Lu et al. (2016) [5]. For whole plastomes, non-coding sequences and ITS datasets, we used their nucleotide sequences directly and aligned each dataset using MAFFT v.7 [33] and trimal [34]. The later software was implemented for alignment trimming. Afterwards, we used Perl scripts to delete the gaps of aligned sequences. For the PCGs dataset, we first extracted 76 PCGs shared by all 33 *Ostrya* plastomes and *Corylus chinensis* and translated their nucleotide sequences into amino acid (aa) sequences for phylogenetic analysis in order to reduce the tree artifacts due to high DNA divergence. Multiple alignment of aa sequence of each gene was achieved by T-coffee [35] followed by trimal [34] to do automated alignment trimming. After that, FASconCAT-G [36] was used to produce a concatenated alignment.

Phylogenetic analyses were performed by both maximum likelihood (ML) and Bayesian inference (BI) methods. For whole plastomes, non-coding regions and ITS datasets, GTR + I + G as the best-fit model selected by JModeltest v.2.1.1 [37] was used for both ML and BI analyses, which were conducted by RAxML v8.1.24 [38] and MrBayes 3.2.2 [39] respectively. For aa sequences of PCGs, Prottest v.3.4.2 [40] was applied to select best-fit aa substitution model for each gene. The selected best-fit models were listed in Additional file 3: Table S3. Then we performed ML analysis using RAxML v8.1.24 with specific aa substitution matrix for each partition by using the selected best-fit aa substitution model. As for BI analysis using MrBayes, preliminary searches (mcmc nchains = 1; ngen = 10,000,000) were made under mixed aamodel (preset aamodelpr = mixed) to identify the best-fit aa substitution model. The result showed that Cprev was the best-fit aa substitution model. Then a final BI analysis using Cprev as the fixed model for all partitions (prset aamodel = fixed(Cprev)) and unlinked model parameters for each partition were conducted in MrBayes.

The node supports were determined with 1000 bootstrap replicates in ML analyses. For BI analysis, two independent parallel runs and four chains (one cold and three hot) were running for 10,000,000 generations with trees sampled every 500 generations. Then we determined convergence by examining trace plots of the log likelihood values for each parameter in Tracer [41]. In addition, we calculated the distances among plastomes of the four *Ostrya* species using the web tool GGDC [42] (http://ggdc.dsmz.de/distcalc2.php) to further compare the inter- and intra-specific variation. As individuals from the same population were grouped together in the phylogenetic trees (see Results), only one individual from each population of the four species was selected for this analysis.

## Results

### Plastome features

**Fig. 1:**
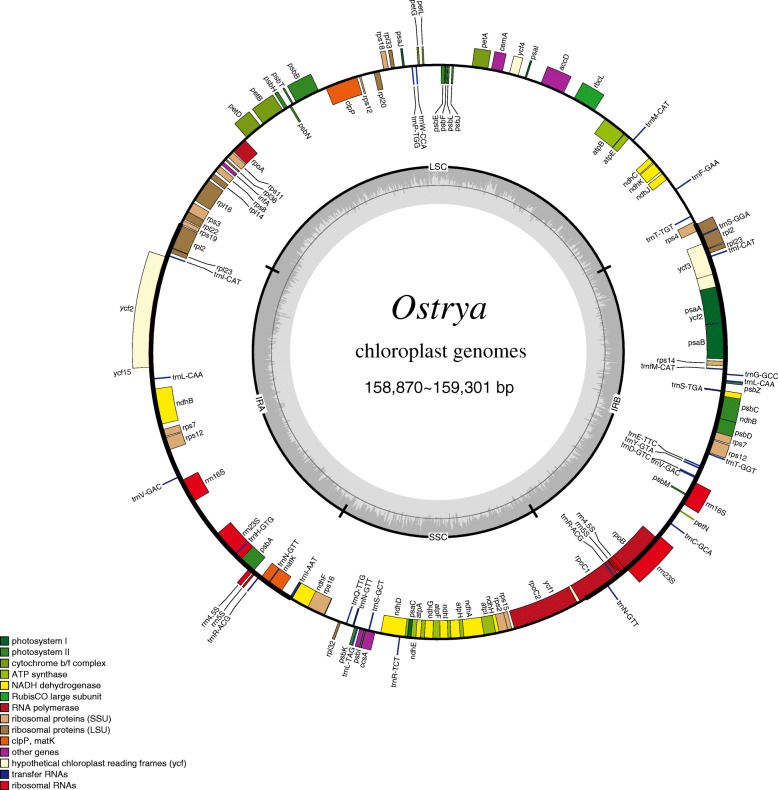
Gene map of the *Ostrya* chloroplast genomes. Because all 33 platomes have identical gene content and gene number, here we only show one plastome map as an example. Genes inside and outside of the circle are transcribed counterclockwise and clockwise directions, respectively. Genes belonging to different functional groups are shown in different colors. The dark gray inner circle corresponds to the GC content and the light-gray circle corresponds to the AT content

**Fig. 2:**
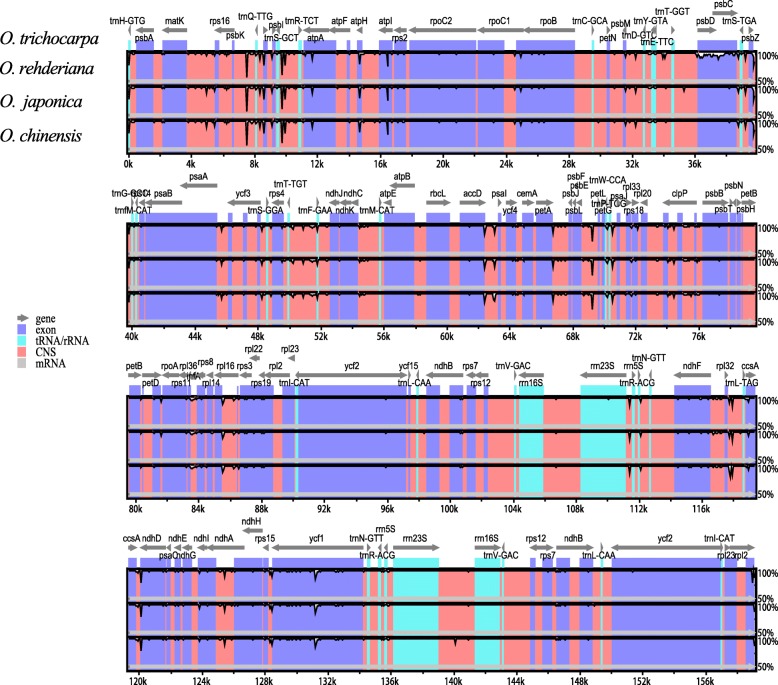
Visualization of alignment of the four *Ostrya* species chloroplast genome sequences using *O. trichocarpa* as the reference. Grey arrows above the alignment indicate the orientation of genes. The vertical scale indicates the percentage of identity, ranging from 50 to 100%. The horizontal axis represents the coordinates within the chloroplast genome. Genome regions are color coded as protein-coding, rRNA coding, tRNA coding, and conserved non-coding sequences, respectively

Characteristics of *Ostrya* plastomes were conservative (Figs. 1 and 2) with the length ranged from 158,870–159,301 bp. The chloroplast genome structures of all the four *Ostrya* species were consistent with mostly known angiosperms with a typical quadripartite structure consisting of a pair of inverted repeats regions (IRa and IRb: 26,059–26,069 bp) divided by a large single-copy region (LSC: 88,007–88,229 bp) and a small single-copy region (SSC: 18,721–18,975 bp) (Fig. 1 and Table 1). The overall GC content was absolutely identical (36.5%; Table 1) across all plastomes. All plastomes possessed 122 unique genes, including 85 protein-coding genes (79 PCG species), 29 tRNA genes (24 tRNA species), and 8 ribosomal RNA genes (4 rRNA species, Table 1). Most genes appeared in a single copy, while 15 were replicated once on the IR regions, including 4 rRNA (4.5S, 5S, 16S, and 23S rRNA), 5 tRNA (*trnI-CAT, trnL-CAA, trnV-GAC, trnR-ACG* and *trnN-GTT*), and 6 PCG species (*rpl2*, *rpl23*, *ycf2*, *ndhB*, *rps7* and *ycf1*; Additional file 4: Table S4). The *rps12* gene was a unique trans-spliced gene with three exons. Expansion of IR regions into rps19 at the IRb/LSC boundary region occurred in all *Ostrya* taxa and the pseudogene *ycf1* was located at the junction of SSC/IRa, which gave rise to the incomplete duplication of protein-coding gene within IRs. In addition, among annotated genes, nine genes (*rps16, atpF, rpoC1, petB, petD, rpl16, rpl2, ndhB* and *ndhA*) contained a single intron, and three genes (*rps12, clpP* and *ycf3*) had two introns. Intergenic spacers showed moderate genetic divergences. The intergenic spacers of *trnQ-psbK*, *trnS-trnR* and *psbZ-trnG* were identified as the most divergence hotspots (Fig. 2).

**Table 1:** Characters of four *Ostrya* species chloroplast genomes

ID	Entire plastid size (bp)	Large single copy (LSC)	Small single copy (SSC)	Inverted repeat (IR)	GC content (%)	Number of genes	Number of protein-coding genes	Number of tRNA genes	Number of rRNA genes
ore01	159,235	88,175	18,944	26,059	36.5	122	85	29	8
ore02	159,218	88,196	18,944	26,059	36.5	122	85	29	8
ore03	159,236	88,176	18,944	26,059	36.5	122	85	29	8
ore04	159,237	88,177	18,944	26,059	36.5	122	85	29	8
oja01	159,241	88,179	18,945	26,059	36.5	122	85	29	8
oja02	159,235	88,186	18,933	26,059	36.5	122	85	29	8
oja06	159,278	88,224	18,933	26,059	36.5	122	85	29	8
oja08	159,280	88,226	18,938	26,059	36.5	122	85	29	8
oja09	159,248	88,180	18,936	26,067	36.5	122	85	29	8
oja10	159,286	88,220	18,930	26,067	36.5	122	85	29	8
oja15	159,250	88,180	18,954	26,059	36.5	122	85	29	8
oja18	159,240	88,180	18,945	26,058	36.5	122	85	29	8
oja19	159,240	88,175	18,944	26,059	36.5	122	85	29	8
oja20	159,229	88,176	18,937	26,059	36.5	122	85	29	8
oja21	159,235	88,177	18,937	26,059	36.5	122	85	29	8
oja23	159,278	88,227	18,937	26,059	36.5	122	85	29	8
oja26	159,238	88,178	18,944	26,059	36.5	122	85	29	8
oja27	159,236	88,169	18,937	26,065	36.5	122	85	29	8
omu01	159,150	88,176	18,954	26,059	36.5	122	85	29	8
omu02	159,251	88,176	18,954	26,059	36.5	122	85	29	8
omu03	159,246	88,176	18,954	26,059	36.5	122	85	29	8
omu08	159,265	88,229	18,917	26,058	36.5	122	85	29	8
omu09	159,227	88,183	18,925	26,058	36.5	122	85	29	8
omu12	159,246	88,176	18,954	26,059	36.5	122	85	29	8
omu13	159,299	88,188	18,956	26,059	36.5	122	85	29	8
omu14	159,288	88,199	18,975	26,059	36.5	122	85	29	8
otr01	159,105	88,118	18,864	26,060	36.5	122	85	29	8
otr03	159,300	88,217	18,966	26,059	36.4	122	85	29	8
otr04	159,301	88,218	18,966	26,059	36.4	122	85	29	8
otr07	159,300	88,219	18,959	26,060	36.4	122	85	29	8
otr08	158,915	88,031	18,743	26,069	36.5	122	85	29	8
otr11	158,879	88,007	18,736	26,069	36.5	122	85	29	8
otr12	158,870	88,008	18,721	26,069	36.5	122	85	29	8

**Fig. 3:**
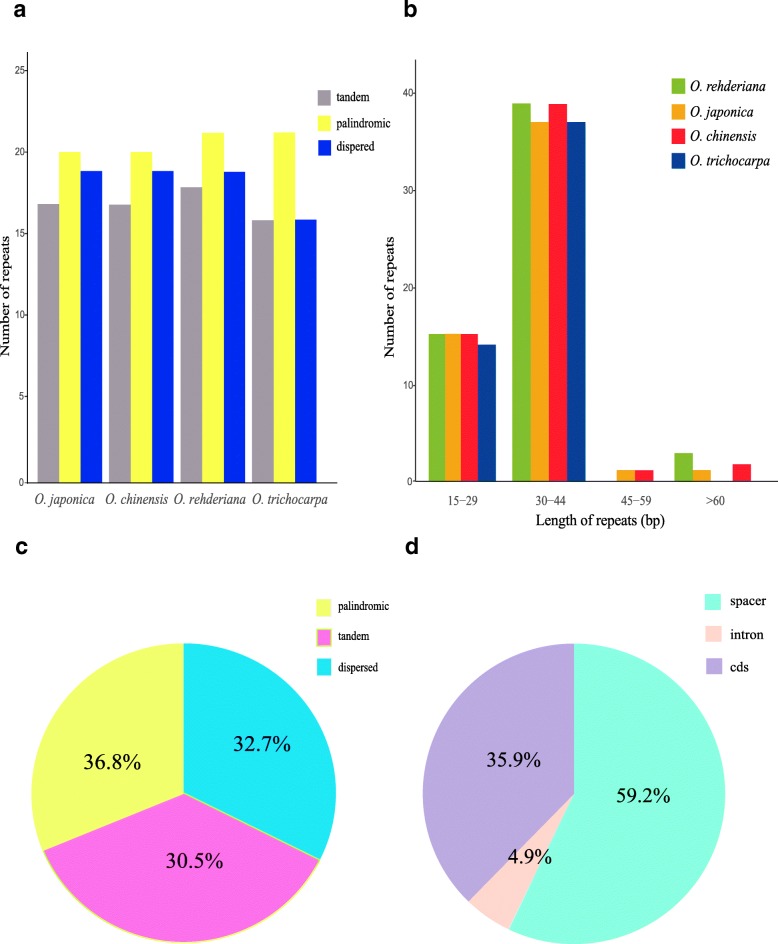
Analysis of repeated sequences in four *Ostrya* plastomes. **a** Number of three repeat types in the four chloroplast genomes; **b** Number of repeated sequences divided by length; **c** Frequency of three repeat types; **d** Frequency of repeats in different regions

Repeat sequences are considered to play a significant role in phylogenetic analysis and also make positive efficiency for genome rearrangement analysis [43, 44]. In this study, a total of 249 repeats were detected in *Ostrya* plastomes (Fig. 3 and Additional file 5: Table S5). All repeats were similar among the four species and their overall distributions were conserved (Fig. 3a). The length of repeated sequences was mainly concentrated from 30 bp to 44 bp (Fig. 3b). The number of the three kinds of repeats was also similar, with palindromic repeat exhibiting maximum amounts (36.4%) followed by dispersed (32.4%) and tandem (31.2%) types (Fig. 3c). Most repeats were detected in intergenic regions (57.1%), while only a minority of repeats were distributed in intron regions (5.2%). The remaining repeats (37.7%) were found in coding regions (e.g., *ycf1, ycf2, atpA, rpl2, rpl23, rps19, rps7, rps12* and *ndhB*; Fig. 3d).

### Phylogenetic analysis

Three plastome datasets (i.e. whole plastomes, PCGs and non-coding regions) and one nuclear ITS sequences were used in this study for phylogenetic analyses. The whole plastome dataset comprised 157,116 bp, 2209 of which were variable and 456 were parsimony-informative. The non-coding dataset comprised 64,479 bp, 1332 of which were variable and 244 were parsimony-informative. 76 PCGs shared by all *Ostrya* samples and outgroup were translated into an amino acid dataset, which contained 21,720 aa, 228 of which were variable and 58 were parsimony-informative. However, the ITS dataset contained 634 bp with only 31 parsimony informative sites (Table 2). It is obvious that the plastome datasets comprised more variable sites than the ITS dataset but the variations rates were far lower than ITS datasets (Table 2).

**Table 2:** Number of informative sites in different datasets

Locus	Whole plastomes (bp)	Protein-coding genes (aa)	Non-coding regions (bp)	ITS (bp)
Constant sites	154,907	21,492	63,147	570
Parsimony informative sites	456	58	244	31
Variable sites	2209	228	1332	64
Total sites	157,116	21,720	64,479	634
Variation rates /%	1.41	1.05	2.07	10.09

For whole plastomes, non-coding regions and ITS datasets, nucleotide sequences were used, while for protein-coding genes, amino acid sequences were used. *bp* base pair, *aa* amino acid

**Fig. 4:**
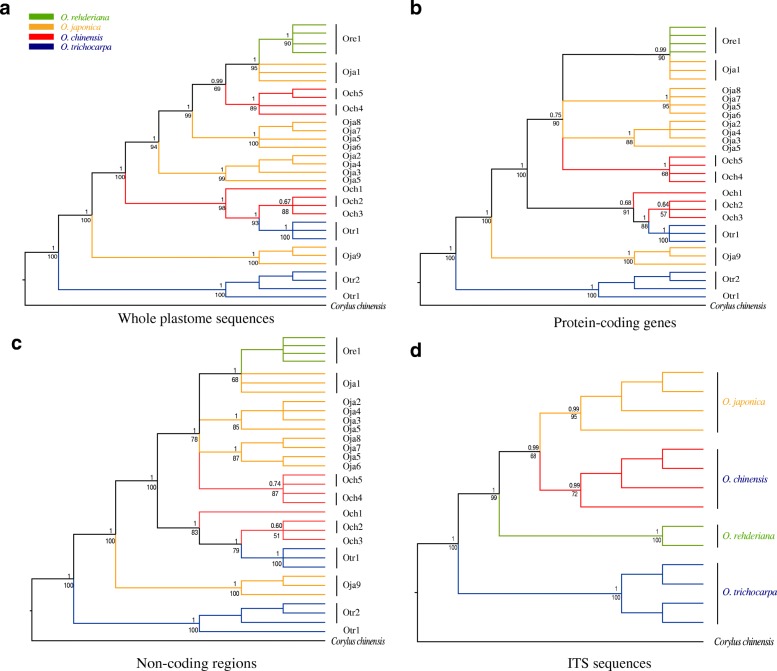
Cladogram of *Ostrya* species using Bayesian inference (BI) analysis and Maximum likelihood (ML) analysis based on different datasets. Support values are shown for nodes as BI posterior probability (above branches) /ML bootstrap (below branches). The four species are shown by different colors. **a** – **c** plastome trees based on different regions; population information for each species is shown at the tips. **d** nuclear ITS (Internal transcribed spacer) tree; samples for each species are selected from different populations

Phylogenetic trees using ML and BI methods done on each dataset resulted in similar topologies and we obtained almost the same topological divergences for all samples based on the PCGs and non-coding region datasets (Fig. 4b and c). The phylogenetic tree derived from the whole plastomes dataset differed slightly from the other two plastome trees. In the former tree, phylogenetic relationships among five subclades (i.e. Ore1, Oja1, Oja2–5, Oja5–8 and Och4–5) were resolved, while relationships among these five subclades were not clear in the later two trees (Fig. 4a, b and c). In general, except for the samples of *O. rehderiana* that collected from the same population, the other three species were all non-monophyletic in the plastome trees (Fig. 4a, b and c). *O. japonica* was separated into four subclades, while the other two species (*O. chinensis* and *O. trichocarpa*) formed another two subclades, respectively. Moreover, some samples that were collected from adjacent regions regardless of species may be clustered together in the plastome trees. For example, population Otr1 of *O. trichocarpa* was geographically close to populations Och2 and Och3 of *O. chinensis* (Additional file 6: Figure S1), and three samples of Otr1 were grouped with samples from populations Och2 and Och3. The heatmap of plastome distances was consistent with such failure of delimitation of interspecific relationships (Additional file 7: Figure S2). However, the ITS tree contrasted greatly with that inferred from the plastomes and the four species were all well-delimitated with high support values in the ITS phylogenetic tree (Fig. 4d).

## Discussion

In the present study, we determined whole plastid genomes of four *Ostrya* species, which provides significant genetic resources and facilitate to comprehend the plastid genome evolution. The exhibition of typical angiosperm quadripartite structure and highly conserved gene content, gene order and GC content in *Ostrya* plastomes were in accord with other members of Betulaceae [45–49]. The presence of repeats, especially in intergenic spacers, was often found associated with divergence regions [50, 51]. We found 57% of repeats occurred in intergenic spacers, which may play a role in maintaining interspecific divergence. In addition, the non-coding regions showed higher interspecific variations than the protein-coding regions as also found in many other groups, and phylogenetic analyses based on different regions may show inconsistence [19, 52], The conservatism of the *Ostrya* plastomes and well-aligned plastomes across different species can therefore facilitate the further phylogenetic analyses.

Previous studies based on a few cpDNA markers failed to resolve the interspecific relationships of *Ostrya* [53, 54], and surprisingly, our analyses based on three different plastome datasets (i.e. whole plastomes, PCGs and non-coding regions) with obviously more informative sites still failed to do so, which is contrast to most plastome studies that recovered high phylogenetic resolution and resolved relationships [55–58]. However, it should be noted that multiple individuals from different populations per species were included in our analyses, and these samples seem to be clustered by geography other than species (Fig. 4, Additional file 6: Figure S1). These results further indicate that phylogenetic studies based on plastome using only one individual per species are not appropriate, especially among closely related species. For example, using one individual from either population Oja9 or Oja1 of *O. japonica* will lead to very different interspecific relationships to other species (Fig. 4).

By contrast, ITS sequences with fewer informatic sites compared with the plastome datasets (Table 2) successfully discriminate the four species. The incongruence between nuclear ITS and cpDNA trees is common observed in many other plants [9, 59, 60] and usually can be explained as follows: firstly, the nuclear ITS sequence has higher mutation rate than chloroplast genome [61] and is biparental inherited with both pollen and seeds dispersion, while chloroplast of most angiosperm is maternal inherited and dispersed only by seeds [62, 63]. Chloroplast DNA with relatively low rates of intraspecific gene flow should be more introgressed, which lead to transfer of genetic material across species boundaries [64]. This may explain why some samples belonging to different species collected from adjacent regions are grouped together in the plastome trees (Fig. 4). In contrast, nuclear loci that experience high rates of intraspecific gene flow should enhance species delimitation [65]. We find the same pattern in our results that the ITS tree has much clearer species delimitation than the plastome trees (Fig. 4). However, alternative scenario may occur in other systems, for example, in *Orychophragmus* [66] and in *Primula* section *Armerina* [67], where cpDNA was highly effective in discriminating closely related species, but nrDNA failed. In addition, incomplete lineage sorting occurred during the fast-radiative speciation of this genus may also play a role in the cyto-nuclear discordance [60, 68]. However, our data is not appropriate to clearly discriminate among these possible scenarios, we therefore recognize that further studies involving whole genome sequences at the population level are needed to better understand their evolutionary histories.

## Conclusions

Our phylogenetic analyses based on plastome datasets still failed to resolve the interspecific delimitation and relationships among four closely related *Ostrya* species, in contrast to the well-resolved phylogeny based on the ITS sequence variations. Such incongruence may result from incomplete lineage sorting and hybrid introgression during the divergences of the four species. Previous plastome studies using one individual per species usually recovered high phylogenetic resolution and resolved relationships, however, our results indicated that multiple samples from different populations should be considered when doing such phylogenetic studies, especially among closely related species. Finally, our findings shed light on some interesting evolutionary questions, e.g., what causes *O. japonica* split into four subclades in the plastome trees, and further genomic studies at the population level are necessary to gain a deep understanding of the evolution of these species.

## Additional files

Additional file 1: **Table S1.** Sample information of Ostrya populations. (DOCX 25 kb)

Additional file 2: **Table S2.** 33 complete chloroplast genomes of four *Ostrya* species from GenBank. (DOCX 17 kb)

Additional file 3: **Table S3.** Best-fit models for each of the amino acid sequence of PCGs. (DOCX 27 kb)

Additional file 4: **Table S4.** Gene list of plastomes of four *Ostrya* species. (DOCX 21 kb)

Additional file 5: : **Table S5.** Repeats distribution in *Ostrya* chloroplast genome. (XLSX 17 kb)

Additional file 6: **Figure S1.** Sample information of *Ostrya* populations. Different dot color indicates the different taxa. Green: *O. rehderiana*. Orange: *O. japonica*. Red: *O. chinensis*. Blue: *O. trichocarpa*. The permission of the graph is not required. (PDF 9547 kb)

Additional file 7: **Figure S2.** Heatmap of plastome distances among 17 *Ostrya* populations. For populations that have multiple samples, we only select one sample in this analysis. (PDF 375 kb)

## Abbreviations

aa: Amino acid
BI: Bayesian inference
bp: Base pair
cpDNA: chloroplast DNA
GC: Guanine cytosine
IR: Inverted repeat region
ITS: Internal transcribed spacer
LSC: Large single copy region
ML: Maximum likelihood
PCGs: Protein coding genes
SSC: Small single copy region
