# Supplementary material for: Interspecific delimitation and relationships among four Ostrya species based on plastomes
Source: BMC Genet. 2019 Mar 12;20:33. doi: 10.1186/s12863-019-0733-0 (PMC6417023; doi:10.1186/s12863-019-0733-0)
Supplement: Supplementary file 1 — Table S1. Sample information of Ostrya populations. (DOCX 25 kb) [file 12863_2019_733_MOESM1_ESM.docx]

**Additional file 1: Table S1.** Sample information of *Ostrya* populations

| **ID** | **Population** | **Species** | **Location** | **Latitude（N）** | **Longitude（E）** | **Altitude（m）** |
| --- | --- | --- | --- | --- | --- | --- |
| ore01 | Ore1 | *Ostrya rehderiana* | Linan,Zhejiang | 30°30’ | 119°45' | 370 |
| ore02 | Ore1 | *Ostrya rehderiana* | Linan,Zhejiang | 30°30’ | 119°45' | 370 |
| ore03 | Ore1 | *Ostrya rehderiana* | Linan,Zhejiang | 30°30’ | 119°45' | 370 |
| ore04 | Ore1 | *Ostrya rehderiana* | Linan,Zhejiang | 30°30’ | 119°45' | 370 |
| oja01 | Oja5 | *Ostrya japonica* | Hongping, Hubei | 31°68’ | 110°44' | 1718 |
| oja02 | Oja5 | *Ostrya japonica* | Hongping, Hubei | 31°68’ | 110°44' | 1718 |
| oja06 | Oja6 | *Ostrya japonica* | Ningshan, Shaanxi | 33°34’ | 108°29‘ | 1638 |
| oja08 | Oja8 | *Ostrya japonica* | Zhouqu, Gansu | 33°35’ | 104°21' | 1772 |
| oja09 | Oja9 | *Ostrya japonica* | Pingwu, Sichuan | 32°38' | 104°31' | 1410 |
| oja10 | Oja9 | *Ostrya japonica* | Pingwu, Sichuan | 32°38' | 104°31' | 1410 |
| oja15 | Oja2 | *Ostrya japonica* | Qinshui, Shanxi | 35°26' | 112°01' | 1589 |
| oja18 | Oja4 | *Ostrya japonica* | Lushi, Henan | 33°45' | 110°50' | 1534 |
| oja19 | Oja1 | *Ostrya japonica* | Huoshan, Anhui | 31°08' | 116°11' | 985 |
| oja20 | Oja1 | *Ostrya japonica* | Huoshan, Anhui | 31°08' | 116°11' | 985 |
| oja21 | Oja1 | *Ostrya japonica* | Huoshan, Anhui | 31°08' | 116°11' | 985 |
| oja23 | Oja7 | *Ostrya japonica* | Tianshui, Gansu | 34°19' | 106°12' | 1526 |
| oja26 | Oja3 | *Ostrya japonica* | Luanchuan, Henan | 33°48' | 111°20' | 1386 |
| oja27 | Oja9 | *Ostrya japonica* | Pingwu, Sichuan | 32°38' | 104°31' | 1410 |
| och01 | Och1 | *Ostrya chinensis* | Luquan,Yunnan | 26°14' | 102°69' | 2392 |
| och02 | Och5 | *Ostrya chinensis* | Wencheng, Zhejiang | 27°87' | 119°84' | 610 |
| och03 | Och5 | *Ostrya chinensis* | Wencheng, Zhejiang | 27°87' | 119°84' | 610 |
| och08 | Och2 | *Ostrya chinensis* | Yinjiang, Guizhou | 27°92' | 108°65' | 1253 |
| och09 | Och2 | *Ostrya chinensis* | Yinjiang, Guizhou | 27°92' | 108°65' | 1253 |
| och12 | Och4 | *Ostrya chinensis* | Longquan,Zhejiang | 30°05' | 121°16' | 1510 |
| och13 | Och4 | *Ostrya chinensis* | Longquan,Zhejiang | 30°05' | 121°16' | 1510 |
| och14 | Och3 | *Ostrya chinensis* | Xinning，Hunan | 26°36' | 111°5' | 1250 |
| otr01 | Otr1 | *Ostrya trichocarpa* | Libo, Guizhou | 25°18' | 107°56' | 956 |
| otr03 | Otr1 | *Ostrya trichocarpa* | Libo, Guizhou | 25°18' | 107°56' | 956 |
| otr04 | Otr1 | *Ostrya trichocarpa* | Libo, Guizhou | 25°18' | 107°56' | 956 |
| otr07 | Otr1 | *Ostrya trichocarpa* | Libo, Guizhou | 25°18' | 107°56' | 956 |
| otr08 | Otr2 | *Ostrya trichocarpa* | Napo, Guangxi | 23°15' | 105°35' | 1260 |
| otr11 | Otr2 | *Ostrya trichocarpa* | Napo, Guangxi | 23°15' | 105°35' | 1260 |
| otr12 | Otr2 | *Ostrya trichocarpa* | Napo, Guangxi | 23°15' | 105°35' | 1260 |
