# Supplementary material for: Interspecific delimitation and relationships among four Ostrya species based on plastomes
Source: BMC Genet. 2019 Mar 12;20:33. doi: 10.1186/s12863-019-0733-0 (PMC6417023; doi:10.1186/s12863-019-0733-0)
Supplement: Supplementary file 2 — Table S2. 33 complete chloroplast genomes of four Ostrya species from GenBank. (DOCX 17 kb) [file 12863_2019_733_MOESM2_ESM.docx]

**Additional file 2: Table S2.** 33 complete chloroplast genomes of four *Ostrya* species from GenBank.

| Species | GenBank accessions |
| --- | --- |
| *Ostrya trichocarpa12 (otr12)* | MG662125 |
| *Ostrya trichocarpa11 (otr11)* | MG662126 |
| *Ostrya trichocarpa08 (otr08)* | MG662127 |
| *Ostrya trichocarpa07 (otr07)* | MG662128 |
| *Ostrya trichocarpa04 (otr04)* | MG662129 |
| *Ostrya trichocarpa03 (otr03)* | MG662130 |
| *Ostrya trichocarpa01 (otr01)* | MG662131 |
| *Ostrya rehderiana04 (ore04)* | MG662132 |
| *Ostrya rehderiana03 (ore03)* | MG662133 |
| *Ostrya rehderiana02 (ore02)* | MG662134 |
| *Ostrya rehderiana01 (ore01)* | MG662135 |
| *Ostrya japonica27 (oja27)* | MG662136 |
| *Ostrya japonica26 (oja26)* | MG662137 |
| *Ostrya japonica23 (oja23)* | MG662138 |
| *Ostrya japonica21 (oja21)* | MG662139 |
| *Ostrya japonica20 (oja20)* | MG662140 |
| *Ostrya japonica19 (oja19)* | MG662141 |
| *Ostrya japonica18 (oja18)* | MG662142 |
| *Ostrya japonica15 (oja15)* | MG662143 |
| *Ostrya japonica09 (oja09)* | MG662144 |
| *Ostrya japonica08 (oja08)* | MG662145 |
| *Ostrya japonica06 (oja06)* | MG662146 |
| *Ostrya japonica02 (oja02)* | MG662147 |
| *Ostrya japonica01 (oja01)* | MG662148 |
| *Ostrya chinensis14 (och14)* | MG662149 |
| *Ostrya chinensis13 (och13)* | MG662150 |
| *Ostrya chinensis12 (och12)* | MG662151 |
| *Ostrya chinensis09 (och09)* | MG662152 |
| *Ostrya chinensis03 (och03)* | MG662153 |
| *Ostrya chinensis02 (och02)* | MG662154 |
| *Ostrya chinensis01 (och01)* | MG662155 |
| *Ostrya chinensis08 (och08)* | MG662156 |
| *Ostrya japonica10 (och10)* | MG662157 |
