# Supplementary material for: Interspecific delimitation and relationships among four Ostrya species based on plastomes
Source: BMC Genet. 2019 Mar 12;20:33. doi: 10.1186/s12863-019-0733-0 (PMC6417023; doi:10.1186/s12863-019-0733-0)
Supplement: Supplementary file 3 — Table S3. Best-fit models for each of the amino acid sequence of PCGs. (DOCX 27 kb) [file 12863_2019_733_MOESM3_ESM.docx]

**Additional file 3: Table S3.** Best-fit models for each of the amino acid sequence of PCGs.

| Gene | Amino acid | | | |
| --- | --- | --- | --- | --- |
| accD | AIC | HIVb+F | BIC | FLU |
| atpA | AIC | HIVw+F | BIC | LG |
| atpB | AIC | RtREV+F | BIC | CpREV |
| atpE | AIC | HIVb | BIC | HIVb |
| atpF | AIC | MtMam+F | BIC | LG |
| atpH | AIC | JTT+F | BIC | CpREV |
| atpI | AIC | CpREV | BIC | CpREV |
| ccsA | AIC | FLU+I+F | BIC | MtArt+I |
| cemA | AIC | HIVb+F | BIC | MtArt |
| clpP | AIC | LG | BIC | LG |
| matK | AIC | HIVw+F | BIC | CpREV |
| ndhA | AIC | HIVb+F | BIC | CpREV |
| ndhB | AIC | MtArt | BIC | MtArt |
| ndhC | AIC | JTT+F | BIC | MtArt |
| ndhD | AIC | JTT+F | BIC | MtArt |
| ndhE | AIC | CpREV | BIC | CpREV |
| ndhG | AIC | MtArt+I | BIC | MtArt+I |
| ndhH | AIC | JTT+F | BIC | CpREV |
| ndhI | AIC | Blosum62 | BIC | Blosum62 |
| ndhJ | AIC | CpREV | BIC | CpREV |
| ndhK | AIC | FLU | BIC | FLU |
| petA | AIC | HIVw+F | BIC | VT |
| petD | AIC | HIVb+F | BIC | CpREV |
| petG | AIC | CpREV | BIC | CpREV |
| petL | AIC | MtMam | BIC | MtMam |
| petN | AIC | MtArt | BIC | MtArt |
| psaA | AIC | HIVb+F | BIC | CpREV |
| psaB | AIC | MtArt+F | BIC | CpREV |
| psaC | AIC | DCMut | BIC | DCMut |
| psaI | AIC | MtArt | BIC | MtArt |
| psaJ | AIC | MtMam | BIC | MtMam |
| psbA | AIC | JTT+F | BIC | CpREV |
| psbB | AIC | MtMam+F | BIC | CpREV |
| psbC | AIC | HIVw+F | BIC | CpREV |
| psbD | AIC | MtREV+F | BIC | CpREV |
| psbE | AIC | CpREV | BIC | CpREV |
| psbF | AIC | CpREV | BIC | CpREV |
| psbH | AIC | MtREV | BIC | MtREV |
| psbI | AIC | MtArt | BIC | MtArt |
| psbJ | AIC | MtArt | BIC | MtArt |
| psbK | AIC | MtArt | BIC | MtArt |
| psbL | AIC | MtArt | BIC | MtArt |
| psbM | AIC | MtREV | BIC | MtREV |
| psbN | AIC | JTT | BIC | JTT |
| psbT | AIC | CpREV | BIC | CpREV |
| psbZ | AIC | MtArt | BIC | MtArt |
| rbcL | AIC | WAG | BIC | WAG |
| rpl14 | AIC | LG | BIC | LG |
| rpl16 | AIC | CpREV | BIC | CpREV |
| rpl2 | AIC | JTT+F | BIC | CpREV |
| rpl20 | AIC | MtMam+F | BIC | CpREV |
| rpl23 | AIC | CpREV | BIC | CpREV |
| rpl32 | AIC | LG | BIC | LG |
| rpl33 | AIC | HIVw | BIC | HIVw |
| rpl36 | AIC | HIVw | BIC | HIVw |
| rpoA | AIC | LG | BIC | LG |
| rpoB | AIC | HIVw+F | BIC | CpREV |
| rpoC1 | AIC | HIVb | BIC | HIVb |
| rpoC2 | AIC | HIVb+F | BIC | CpREV |
| rps11 | AIC | HIVb+F | BIC | CpREV |
| rps12 | AIC | CpREV | BIC | CpREV |
| rps14 | AIC | HIVb | BIC | HIVb |
| rps15 | AIC | Dayhoff+F | BIC | HIVb |
| rps16 | AIC | Blosum62 | BIC | Blosum62 |
| rps18 | AIC | HIVw+F | BIC | HIVw |
| rps19 | AIC | HIVw | BIC | HIVw |
| rps2 | AIC | Blosum62 | BIC | Blosum62 |
| rps3 | AIC | HIVb | BIC | HIVb |
| rps4 | AIC | HIVb | BIC | HIVb |
| rps7 | AIC | CpREV | BIC | CpREV |
| rps8 | AIC | HIVw | BIC | HIVw |
| ycf1 | AIC | HIVb+I+F | BIC | HIVb+I+F |
| ycf15 | AIC | CpREV | BIC | CpREV |
| ycf2 | AIC | MtMam+F | BIC | MtMam+F |
| ycf3 | AIC | LG | BIC | LG |
| ycf4 | AIC | CpREV | BIC | CpREV |
