# Supplementary material for: Interspecific delimitation and relationships among four Ostrya species based on plastomes
Source: BMC Genet. 2019 Mar 12;20:33. doi: 10.1186/s12863-019-0733-0 (PMC6417023; doi:10.1186/s12863-019-0733-0)
Supplement: Supplementary file 4 — Table S4. Gene list of plastomes of four Ostrya species. (DOCX 21 kb) [file 12863_2019_733_MOESM4_ESM.docx]

**Additional file 4: Table S4.** Gene list of plastomes of four *Ostrya* species.

| **Category for genes** | **Group of genes** | **Name of genes** | | | | |  |  |
| --- | --- | --- | --- | --- | --- | --- | --- | --- |
| **Self-replication** | Ribosomal RNA genes | *rrn16^b^* | *rrn23^b^* | *rrn4.5^b^* | *rrn5^b^* |  |  |  |
|  | Transfer RNA genes | *trnH-GTG* | *trnQ-TTG* | *trnS-GCT* | *trnR-TCT* | *trnC-GCA* |  |  |
|  |  | *trnD-GTC* | *trnY-GTA* | *trnE-TTC* | *trnT-GGT* | *trnS-TGA* |  |  |
|  |  | *trnG-GCC* | *trnfM-CAT* | *trnS-GGA* | *trnT-TGT* | *trnF-GAA* |  |  |
|  |  | *trnM-CAT* | *trnW-CCA* | *trnP-TGG* | *trnI-CAT^b^* | *trnL-CAA^b^* |  |  |
|  |  | *trnV-GAC^b^* | *trnR-ACG^b^* | *trnN-GTT^a,b^* | *trnL-TAG* |  |  |  |
|  | Small subunit of ribosome | *rps12^a^* | *rps16^a^* | *rps2* | *rps14* | *rps4* | *rps18* |  |
|  |  | *rps11* | *rps8* | *rps3* | *rps19* | *rps7^b^* | *rps15* |  |
|  | Large subunit of ribosome | *rpl33* | *rpl20* | *rpl36* | *rpl14* | *rpl16^a^* | *rpl22* | *rpl2^a,b^* |
|  |  | *rpl23^b^* | *rpl32* |  |  |  |  |  |
|  | DNA dependent RNA polymerase | *rpoC2* | *rpoC1^a^* | *rpoB* | *rpoA* |  |  |  |
|  | Translational initiation factor | *infA* |  |  |  |  |  |  |
| **Genes for photosynthesis** | Subunits of photosystem I | *psaB* | *psaA* | *psaI* | *psaJ* | *psaC* |  |  |
|  | Subunits of photosystem II | *psbA* | *psbK* | *psbI* | *psbM* | *psbD* | *psbC* | *psbZ* |
|  |  | *psbL* | *psbF* | *psbE* | *psbB* | *psbT* | *psbN* | *psbH* |
|  |  | *psbJ* |  |  |  |  |  |  |
|  | Subunits of cytochrome | *petN* | *petA* | *petL* | *petG* | *petB^a^* | *petD^a^* |  |
|  | Subunits of ATP synthase | *atpA* | *atpF^a^* | *atpH* | *atpI* | *atpE* | *atpB* |  |
|  | ATP-dependent protease subunit p gene | *clpP^a^* |  |  |  |  |  |  |
|  | Large subunit of Rubisco | *rbcL* |  |  |  |  |  |  |
|  | Subunits of NADH dehydrogenase | *ndhJ* | *ndhK* | *ndhC* | *ndhB^a,b^* | *ndhF* | *ndhD* | *ndhE* |
|  |  | *ndhG* | *ndhI* | *ndhA^a^* | *ndhH* |  |  |  |
| **Other genes** | Maturase | *matK* |  |  |  |  |  |  |
|  | Envelop membrane protein | *cemA* |  |  |  |  |  |  |
|  | Subunit of acetyl-CoA-carboxylase | *accD* |  |  |  |  |  |  |
|  | c-type cytochrome synthesis gene | *ccsA* |  |  |  |  |  |  |
| **Genes of unknown function** | Conserved open reading frames | *ycf3^a^* | *ycf4* | *ycf2^b^* | *ycf15* | *ycf1* |  |  |

^a^ Gene contains introns; ^b^ Gene with two copies.
