# Supplementary figures and images for: Interspecific delimitation and relationships among four Ostrya species based on plastomes
Source: BMC Genet. 2019 Mar 12;20:33. doi: 10.1186/s12863-019-0733-0 (PMC6417023; doi:10.1186/s12863-019-0733-0)

0 500 1,000 Km

Legend

exdem2

Value

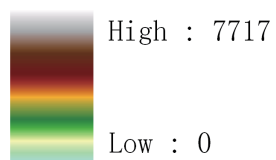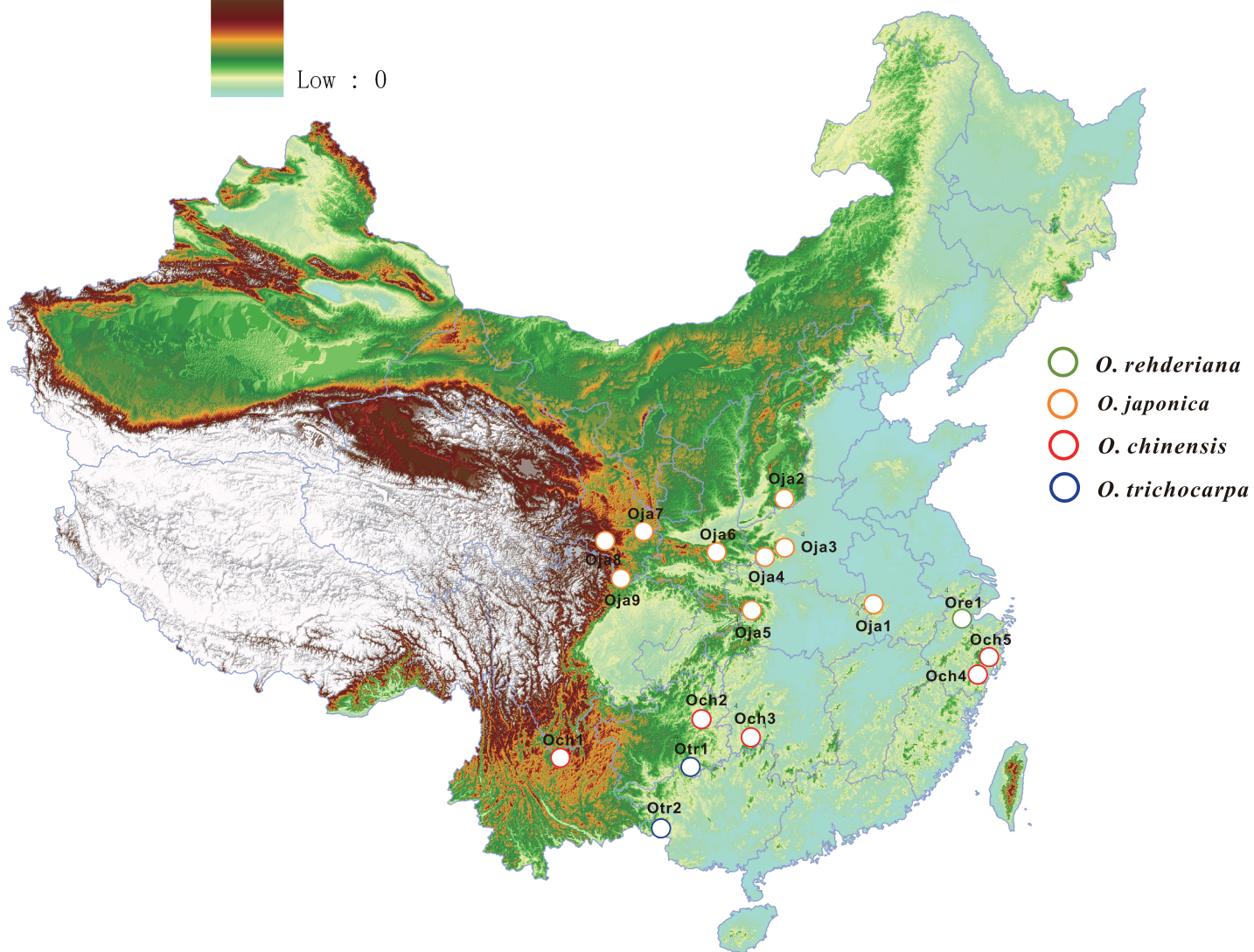

Supplement: Supplementary file 6 — Figure S1. Sample information of Ostrya populations. Different dot color indicates the different taxa. Green: O. rehderiana. Orange: O. japonica. Red: O. chinensis. Blue: O. trichocarpa. The permission of the graph is not required. (PDF 9547 kb) [file 12863_2019_733_MOESM6_ESM.pdf]

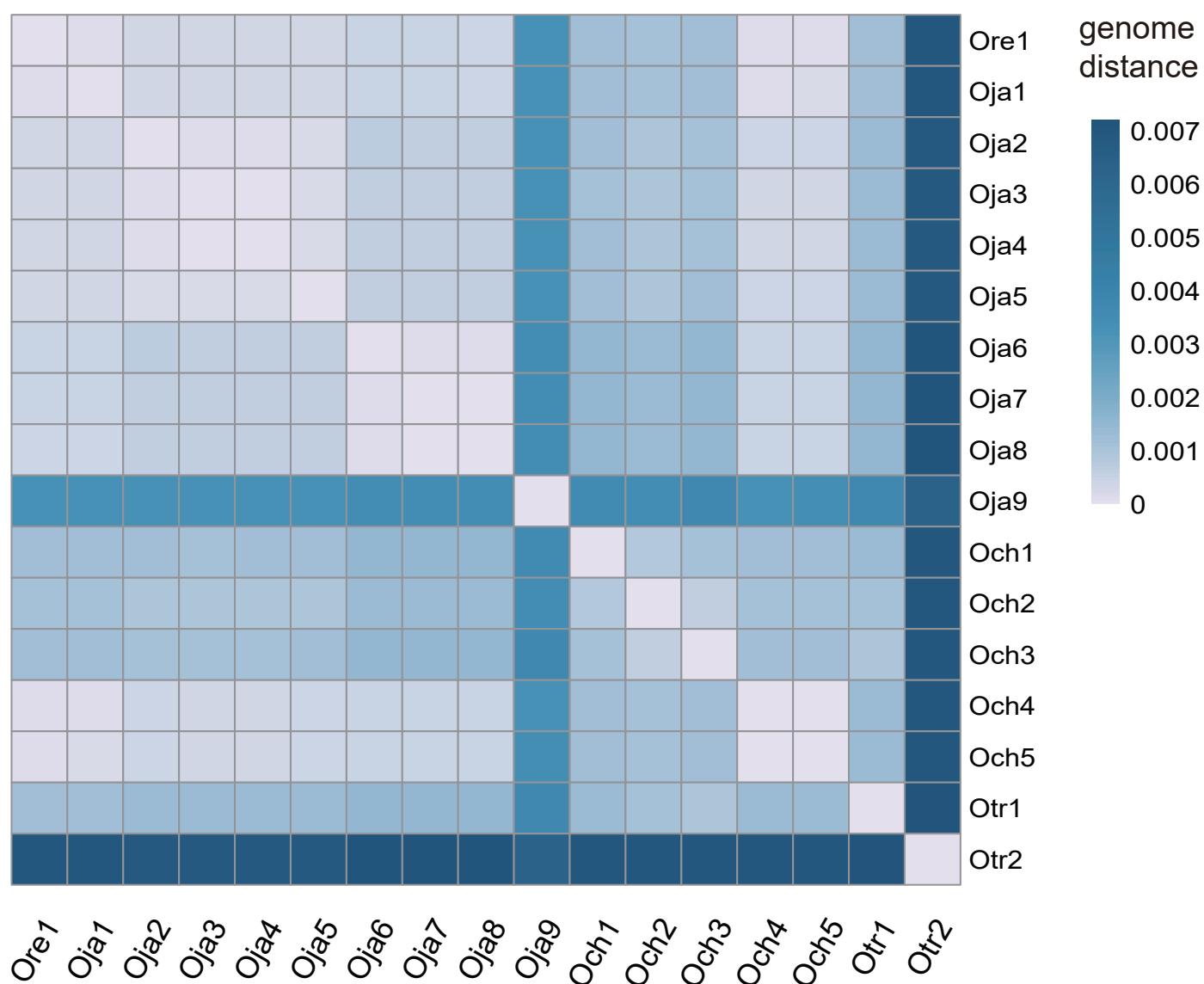

Supplement: Supplementary file 7 — Figure S2. Heatmap of plastome distances among 17 Ostrya populations. For populations that have multiple samples, we only select one sample in this analysis. (PDF 375 kb) [file 12863_2019_733_MOESM7_ESM.pdf]
